# Comparative transport analysis of cell penetrating peptides and Lysosomal sequences for selective tropism towards RPE cells

**DOI:** 10.21203/rs.3.rs-3651531/v1

**Published:** 2023-12-27

**Authors:** Kris Grohn, Kyle Parella, Ellie Lumen, Hanna Colegrove, Victor Bjork, Alana Franceski, Aaron Wolfe, Kelsey Moody

**Affiliations:** SUNY-ESF: SUNY College of Environmental Science and Forestry; Ichor Life Sciences, Inc; Ichor Life Sciences, Inc.; Ichor Life Sciences, Inc.; Ichor Life Sciences, Inc.; Ichor Life Sciences, Inc.; Ichor Life Sciences, Inc.; Ichor Life Sciences, Inc.

**Keywords:** Cell penetrating peptides, Retinal biologics drug delivery, Subcellular delivery, retinal pigment epithelium

## Abstract

Cell penetrating peptides are typically nonspecific, targeting multiple cell types without discrimination. However, subsets of Cell penetrating peptides (CPP) have been found, which show a ‘homing’ capacity or increased likelihood of internalizing into specific cell types and subcellular locations. Therapeutics intended to be delivered to tissues with a high degree of cellular diversity, such as the intraocular space, would benefit from delivery using CPP that can discriminate across multiple cell types. Lysosomal storage diseases in the retinal pigment epithelium (RPE) can impair cargo clearance, leading to RPE atrophy and blindness. Characterizing CPP for their capacity to effectively deliver cargo to the lysosomes of different cell types may expand treatment options for lysosomal storage disorders. We developed a combinatorial library of CPP and lysosomal sorting signals, applied to ARPE19 and B3 corneal lens cells, for the purpose of determining cell line specificity and internal targeting. Several candidate classes of CPP were found to have as much as 4 times the internalization efficiency in ARPE19 compared to B3. Follow-up cargo transport studies were also performed, which demonstrate effective internalization and lysosomal targeting in ARPE19 cells.

## Introduction

The abnormal accumulation of material in the lysosome is the cause of a broad class of diseases termed lysosomal storage disorders. These disorders are typically caused by deficient activity in lysosomal enzymes, such as seen in mutations of GBA1 leading to Gaucher’s disease which is characterized by accumulation of glucocerebroside lipids.^1^ The accumulation of metabolic byproducts over long periods is also a factor in promoting these diseases, such as seen in lipofuscin formation in aged cells and the accumulation of bisretinoid molecules in the RPE cell layer of the eye which is associated with the onset and progression of Stargardt’s disease and AMD^2,3^. Enzyme replacement therapy is one therapeutic modality for removing accumulated lysosomal material. In Guacher’s disease treatments, and in some experimental methods for treating macular degeneration, this is accomplished through the delivery of therapeutic proteins via mannose-receptor mediated endocytosis^4^. However, the difficulties of protein glycoengineering, as well as the inefficiency and non-specificity of glycan delivery system has driven research into targeted enzyme delivery systems (EDSs)^5–8^.

CPP have become popular EDSs for therapeutic enzymes since their discovery in 1988, when it was observed that the trans-activator protein (Tat) of HIV-1 allowed for translocation across cell membranes into the cytoplasm^9–12^. Besides the capacity for translocation across membranes, CPP can perform a variety of novel functions due to the diversity in their sequence, electrostatic charge, hydrophobicity, and structure. Certain CPP have demonstrated a “Homing” capacity, defined as the ability to preferentially penetrate specific cell types such as cancer cell lines, which may enable more efficient delivery and lower off target toxicity^13–15^. CPP have also been found which deliver cargos preferentially to subcellular locations, with successful delivery to the nucleus, endoplasmic reticulum, and lysosomes. Due to these features, the application of CPP for site directed biologics has been rapidly expanding in the field of oncology, where numerous CPP-conjugates have entered clinical development over the last decade^16–18 19^. As CPP research continues to evolve, their application as EDSs may improve the efficacy, safety, and scope of enzyme replacement therapeutics.

Cell line and subcellular targeting CPP may serve as useful EDSs in delivery of therapeutic enzymes to the posterior segment of the retina. The retina of the eye is composed of layers of specialized epithelial and neural cells that serve as a physical barrier between the posterior segment and the intraocular space. Therapeutic enzymes intended for delivery to the cells at the posterior of the eye, such as the retinal pigment epithelium (RPE) are required to pass by this complex layers of cells as they diffuse across the retina^20,21^. Cell line specific CPP may serve as ideal EDSs for targeted delivery to the RPE, which may open new avenues for therapeutic delivery of novel enzymes for the treatment of Stargardt’s disease and AMD.

The focus of this study is to characterize the features of CPP that show higher internalization to the RPE, and to determine whether the inclusion of lysosomal sorting signals would improve localization of a fluorescent cargo to lysosomes. Based on the membrane characteristics of RPE cells and known cell-specific surface receptors, we identified 50 candidate CPPs that we anticipated to show greater internalization into RPE cells^28,29^. To achieve lysosomal targeting, we coupled these 50 candidates with 9 lysosomal targeting motifs, resulting in a library of 500 CPP-LYS constructs. These CPP-LYS constructs were tested in ARPE-19 and B3 cells for their ability to enter the cell and hone to the lysosome, resulting in several candidate sequences with a preference for AREP19 localization that could be applied to future in vivo studies.

## Results

### Uptake differences between cell types

**Figure 1:**
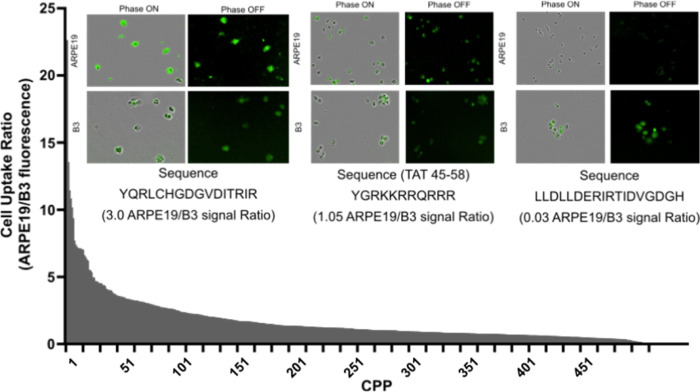
Overview of the distribution of CPP ARPE19/B3 uptake ratio and representative images

**Figure 2:**
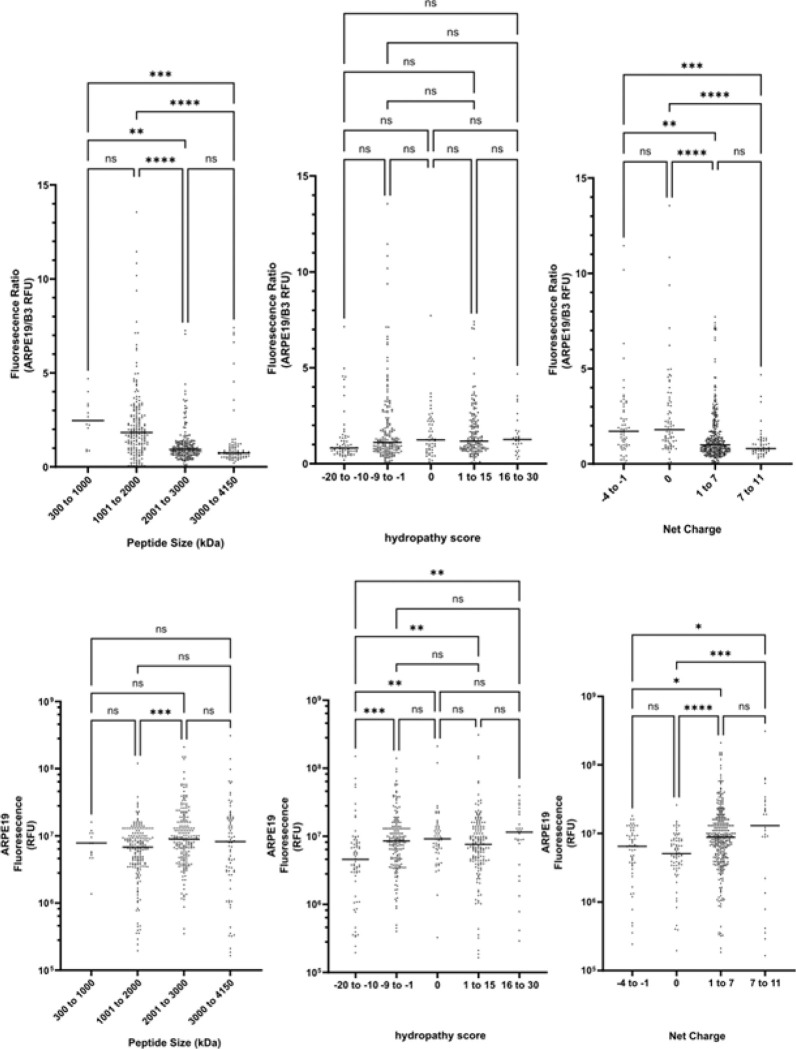
Cell uptake of the CPPs (expressed in fluorescence intensities (RFU) and ARPE19/B3 cell uptake ratio) compared with respect to molecular weight, net charge, and hydropathy score.

Comparative internalization efficiency was measured by comparing green channel fluorescence of treated APRE19 cells to B3 cells(Supplemental Table 2). The ratio of ARPE19 green fluorescence to B3 cell fluorescence was mostly equivalent between cell lines (Fig. 1) with ~ 13.5% of the CPP’s having a ratio ≥ 3.0 (favoring ARPE19 internalization) and 4.0% having a ratio ≤ 0.3 (discriminatory against ARPE19 internalization). The influence of biophysical characteristics on CPP uptake efficiency between cell lines was significant for the overall degree of internalization but did not significantly contribute to improving the ratio of internalization between ARPE19 cells compared to B3 cells. Net charge, hydropathy, and sequence length were found to have a negligible impact on internalization efficiency between cell lines, but Net charge, hydropathy were associated with increased uptake. Sequence length did not significantly impact the rate of CPP uptake and did not contribute to cell line uptake specificity (Fig. 2).

**Figure 3:**
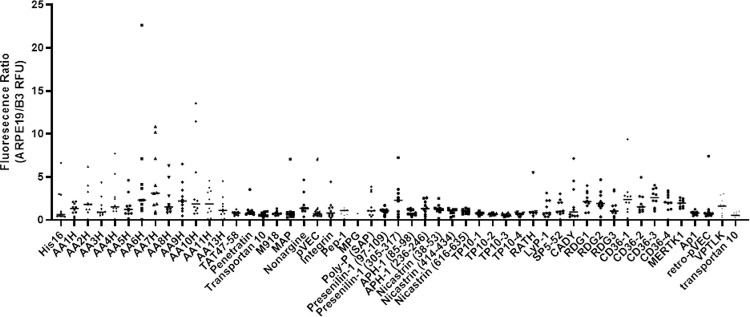
Visual Summary of the ratio of ARPE19 to B3 cell uptake Ratio by CPP identity

**Figure 4:**
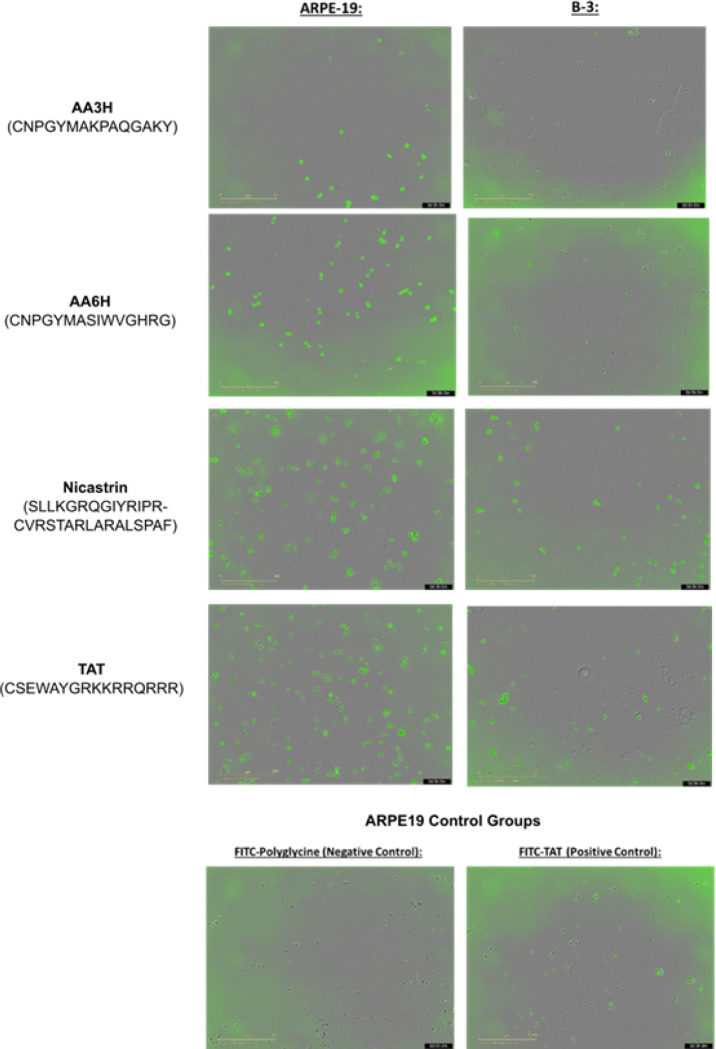
Representative images of the CPP that were selected to move on to cargo transport studies

CPP Class and sequence specificity contributed strongly to both internalization efficacy and cell line specificity (Fig. 3). CPP with the Membrane targeting, metabotropic, and surface receptor general classes averaged a ratio greater than 1.5 (2.4, 2.1, and 1.7 ARPE19/B3 cell internalization efficacy ratio respectively). Specific CPP sequences selected for their capacity to selectively target membranes based on their lipid composition showed the most significant cell line specificity. The AAXH class (X = 1 through 13) of CPP was enriched for ARPE19 targeting, with AA10, AA9H, and AA4H demonstrating a mean uptake ratio of 4.6 ± 2.1, 4.3 ± 1.2, and 4.1 ± 1.5 respectively (Fig. 4). Similarly, the cell surface receptor specific peptides CD36–1 and CD36–3 demonstrated a high mean uptake ratio, preferring ARPE19 to B3 cells at a ratio of 2.7 ± 0.8 and 2.5 ± 0.3 respectively, but it was characteristic of this class that the internalization rate overall was low in comparison to standard CPP.

### Confocal Microscopy Analysis of Lysosomal Localization

**Figure 5:**
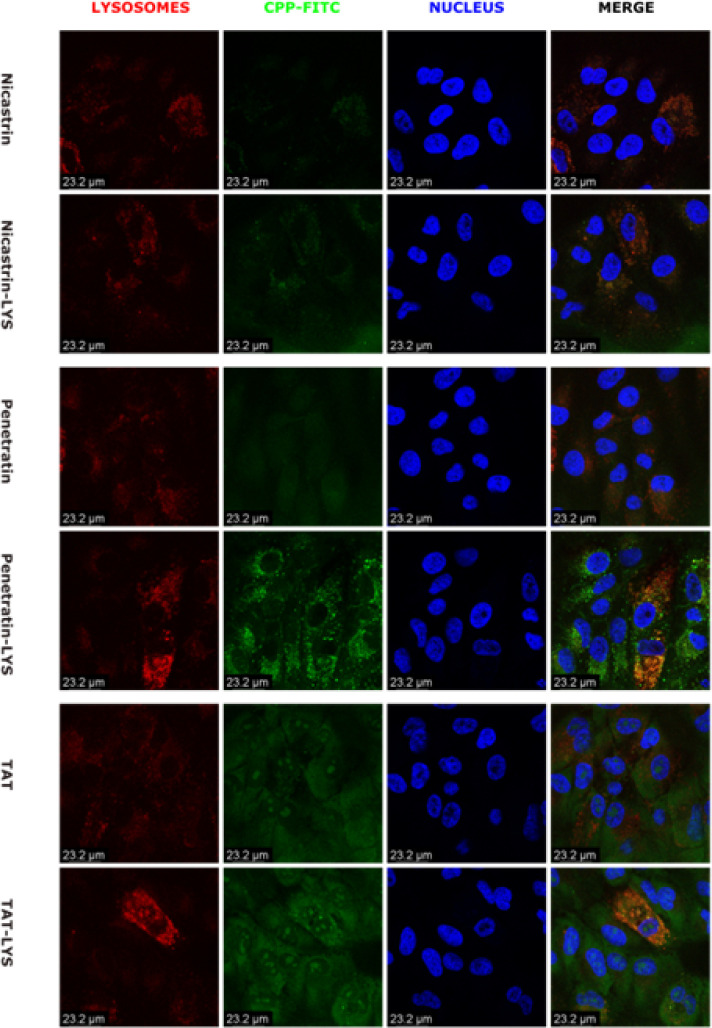
Confocal microscopy analysis of selected lysosomal targeting constructs in ARPE19 cells. 1 μM solutions of each FITC labelled CPP were added to the culture media of ARPE-19 cells for 3 hours before imaging. Each set of images demonstrates the native CPP compared with a modified CPP co-expressed with a lysosomal targeting sequence on its N-terminus. Both sets of constructs were labelled with FITC (Green) and Lysotracker Red (Red), with the nulcei stained with Hoest nuclear Stain (Blue).

**Figure 6:**
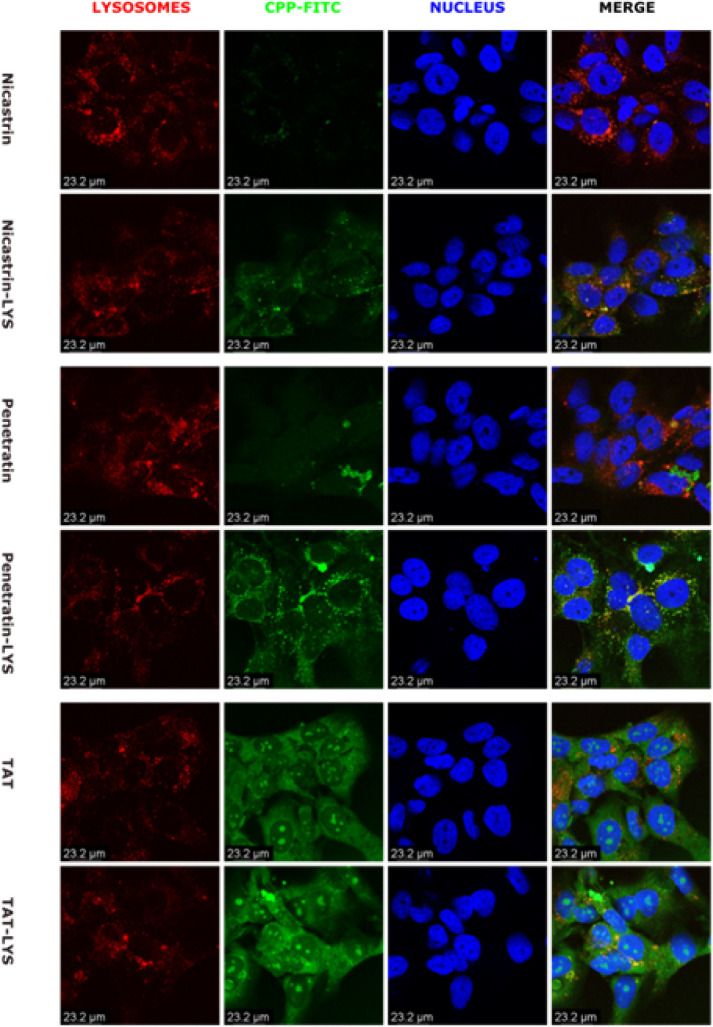
Confocal microscopy analysis of selected lysosomal targeting constructs in B3 cells. 1 μM solutions of each FITC labelled CPP were added to the culture media of ARPE-19 cells for 3 hours before imaging. Each set of images demonstrates the native CPP compared with a modified CPP co-expressed with a lysosomal targeting sequence on its N-terminus. Both sets of constructs were labelled with FITC (Green) and Lysotracker Red (Red), with the nulcei stained with Hoest nuclear Stain (Blue).

3 Representative CPP sequences and their lysosomal sorting counterparts (TAT (YGRKKRRQRRR), Penetratin (RQIKIWFQNRRMKWKK), Nicastrin (RLPRCVRSTARLARALSPAF) were selected for higher magnification confocal microscopy imaging due to desirable features observed in the lower magnification imaging and their relevance in the CPP literature. Each CPP was compared between ARPE19 cells and B3 cells as in the screening study. For the Nicastrin and Penetratin series, the inclusion of a lysosomal targeting tag increased overall green fluorescence and demonstrated and preferentially localized within lysosomes but we also observe a general decrease of red fluorescence from Lysotracker in ARPE19 cells, but not B3 cells. Lysotracker is a pH sensitive dye, so this may indicate deacidification of the lysosomes of the RPE due to membrane disruption, or a reaction specific to the ARPE19 cells in response to a foreign material entering the lysosome. The inclusion of a lysosomal targeting tag in the TAT series of CPP did not reduce distribution through the cytoplasm, but appears to promote higher association with the lysosome. This may reflect a mechanical aspect of the TAT peptide itself, as this sequence is known for escaping endosomes. Like Nicastrin and Penetratin, Lysosome fluorescence was dimmed in ARPE19(Fig. 5), but mostly unaffected in B3 cells (Fig. 6). B3 cells display a higher degree of fluorescence overlap than ARPE19, which may indicate differences due to the composition of their membrane or endosome.

## Discussion

### CPP synthesis and preparation

We used a three prong methodology for selecting potential CPP’s/internalization signals: (i) Database and literature review to select a chemically diverse selection of popular CPP that were known to act through different internalization mechanisms^9,22^. (ii) we selected less common CPP that were known to have a selective internalization efficiency based on the relative lipid composition of the cell membrane, as the RPE membrane is enriched for phosphatidylethanolamine compared to the other cells of the eye. (iii), we selected receptor mediated internalization sequences that were known to bind to surface receptors CD-36, MERTK, and integrin, that are present in ARPE19 cells, which would be the cell culture model used experimentally. We produced a final selection of 50 CPP sequences(Supplementary Table 1), with each of these produced alone or with the CPP sequences appended to 9 different lysosomal targeting sequences. Each sequence was prepared via parallel peptide synthesis with a FITC fluorophore attached to the N terminus of these sequences via a β-alanine linker as a model cargo. Lysosomal targeting sequences were selected from literature sources reporting on the sorting and trafficking of lysosomal proteins.

The aim of this study was to determine whether different classes of cell penetrating peptide could selectively target different lineages of cell within the eye, and to determine whether the addition of lysosomal targeting sequences could be appended to these sequences to allow for cell-line and organelle specific targeting in a single motif. Peptides were selected from different known classes of CPP and for the potential to interact with surface proteins that are enriched on the surface of RPE cells. The CPP library was produced in conjugation with an N terminal Lysosomal targeting sequence, and with an N terminal cap of the fluorophore FITC linked to the peptide with a beta-alanine linker, which prevents self-cleavage of FITC during peptide synthesis^23^. This sequence format was selected to maximize the distance of the fluorophore from the CPP region of each peptide.

B3 Corneal epithelial cells were selected as a comparison cell line for our applications due to their accessibility as an eye cell line, their similar size to ARPE19 cells, and the similarity of culturing conditions to ARPE19^24^. One limitation of this study is that we did not use a photoreceptor cell line to gauge specificity and toxicity in a nerve cell or photoreceptor that would be at the same site of action are the RPE cell layer, due to difficulty of applying this cell line to screening conditions, as they achieve low cell densities than can be achieved compared to ARPE19, which would complicate the comparison, and are quite fragile in culture^25^. Further studies using primary polarized RPE cells and a primary photoreceptor cell line such as 661W would provide a more ideal comparison for physiological conditions.

Our fluorescence detection was performed with an I3 spectrophotometer and a Sartorius IncuCyte S3 automated fluorescence cell monitoring system. The screening plates had fluorescence quantified in both the I3 and Sartorius IncuCyte S3 to provide orthogonal fluorescence screening using a different instrument than is typically used in related literature and for capturing brightfield images. We used I3 fluorescence counts for the graphs displayed, as historically fluorescence studies of CPP are used with raw fluorescence counts on a plate reader format^26^. To improve data quality, future studies might be performed using a high throughput confocal microscope to provide more detailed images of fluorescence overlap in screening and localization studies.

The most successful CPP classes in terms of their internalization ratio of ARPE19 to B3 cells were found to be membrane selective, which supports our hypothesis that the enriched phosphatidylethanolamine layer of RPE (67.5%) compared to the retina (38.6%), lens (15%), or cornea (8.5%) may provide enough of a basis to preferentially deliver CPP’s and their cargo to the RPE cell layer^27,28^. Annexin A isoform derived CPP (AA1H to AA13H) have been shown previously to have lipid binding properties, with annex III, IV, V, and VI preferring phosphatidylethanolamine. In this screen, Annexin III-VII all had a mean ratio greater than 1.5 (ARPE:B3 fluorescence), with AA6H being the highest of this class at a ratio of 4.7^29^. Future studies should be carried out using these classes in comparison with a variety of other cells with known compositions of cell membrane lipids, or artificial defined membranes to verify the mechanism of internalization to be based on the ratio of phosphatidylethanolamine.

## Conclusion

The CPP developed in this screen demonstrate a preference for ARPE19 cells vs B3 corneal cells and can be used to internalize a recombinantly linked cargo protein effectively. Mechanistically, this is likely due to the membrane composition of RPE cells being enriched in phosphatidylethanolamine. As the RPE cell layer is implicated the development of macular degeneration, therapeutic fusion proteins prepared with these cell penetrating peptides may be used to deliver catabolic proteins that could be used to degrade intracellular lipofuscin in the RPE, which may delay the progression of macular degeneration. Other applications might be used for enzyme replacement therapy in other phospholipid rich cell lines.

## Methods

### Peptide synthesis

Peptides were synthesized via automated peptide synthesis using a syro I peptide synthesis robot (Manufacturer). Peptides were assembled on a RINK-amide resin in parallel 96-well tip synthesis format using 10 mg of resin per tip. The resin was primed for synthesis by three cycles of resin incubation with 200 μL dimethylformamide (DMF) for 30 minutes. FMOC deprotection was performed at each stage of synthesis by treating the resin three times with 80 μL of a 40% piperidine/60% DMF mixture then washing the resin 8 times with 100 μL of DMF. Coupling was performed by incubating the resin with 6 molar equivalents of a preactivated mixture of each FMOC protected amino acid for 30 minutes. Coupling steps were performed twice, first using a 2/2/1 mixture of 450 mM FMOC amino acid in DMF/ 450 mM N,N’-Diisopropylcarbodiimide in DMF/ 900 mM Oxyma in DMF, and secondly using a 2/2/1 mixture of 450 mM FMOC amino acid in DMF/430 mM (2-(1H-benzotriazol-1-yl)-1,1,3,3-tetramethyluronium hexafluorophosphate in DMF/ 1.8 M N,N-Diisopropylethylamine in N-Methyl-2-pyrrolidone. Each coupling step was performed using 100 μL of each preactivation mixture, and was followed with washing 4 times with 110 μL of DMF and a deprotection step as described previously. After the final amino acid coupling, two additional rounds of coupling and deprotection were performed to create an N terminal Fluorescein label connected to the peptide chain via a Beta-alanine linker. The beta alanine linker coupling was performed as specified above for the individual amino acid building block steps, and the fluorophore coupling step was performed by incubating the resin overnight in 100 μL of 3 molar equivalents of Fluorescein isothiocyanate isomer I dissolved in a mixture of DMF and 6 molar equivalents of DIPEA followed by washing the resin 8 times with 110 μL DMF. Cleavage of the peptide from the resin backbone was performed by washing the resin with Dichloromethane, then treating the resin for 2 hours with 200 μL of a mixture composed of 92.5% TFA, 2.5% TIS, 2.5% DODT, 2.5% Water. The labeled peptides were collected in a deep well 96 well plate, precipitated using cold ethyl ether and then centrifuged at 1000 RPM for 10 minutes to pellet the precipitate. Pelleted peptides were then resuspended in cold ethyl ether and centrifuged again in a similar fashion for two additional cycles to wash the peptides of residual cleavage cocktail. The peptides were then resuspended in 10 mM HCl, frozen overnight at −80°C, and lyophilized to remove water and trifluoroacetic acid. The resulting peptide powders were sealed and stored at −80°C.

### Adjustment of peptide concentrations

One milliliter of 20% ethanol was added to each well of the 96 well plates containing the lyophilized peptides and the plates were then re-sealed. Sealed plates were placed until halfway submerged into a bath sonicator for 30 minutes to aid resuspension. The plates were centrifuged at 1000 RPM for 10 minutes to pellet any undissolved material. A 100 μL volume of each peptide were transferred into a clear bottom 96 well plate containing an internal standard of FITC in 80% water/20% ethanol, 10 mM HCl. Each peptide was quantified using FITC absorbance as a proxy for peptide concentration. Each peptide was normalized to 50 μM in a new deep well 96 well plate by adding calculated amounts of 20% ethanol and labelled peptide, then aliquoted to opaque black 96 well daughter plates that were sealed with foil and stored at −80°C.

### Cell culture

Human B3 corneal epithelial cells and ARPE19 cells were obtained from ATCC. Cells were grown to confluency using DMEM at 37°C, 5% CO2. ARPE-19 and B3 cells for uptake experiments were seeded into a dark walled, clear bottom 96 well plate at 200 cells per well. All solutions were warmed to 37°C, and CPP incubations were performed at 37°C, 5% CO2.

### CPP uptake

CPP working solutions were prepared by diluting the samples from the 50 μM stock plate to 10 μM in cell growth media. Cells were incubated with 100 μL of the 10 μM CPP solution for 2 hours, after which the CPP solution was aspirated from the wells, which were then washed three times with phosphate buffered saline. Cells were then incubated with 100 uL of PBS for analysis. Raw Cell fluorescence values were monitored using a spectramax I3 plate reader using an excitation wavelength of 495 nm and an emission wavelength of 530 nm. An Incucyte live cell analysis microscopy system was used to extract brightfield and fluorescence images of the cells as well, using a 20X objective microscope for brightfield images and a green fluorescent channel with an excitation wavelength of 460 nm (passband 440,480 nm) and an emission wavelength of 524 nm (passband 504,544 nm). Incucyte software was used to determine green calibrated units and phase area.

### Confocal Microscopy

Three representative sequences: TAT (YGRKKRRQRRR), Penetratin (RQIKIWFQNRRMKWKK), Nicastrin (RLPRCVRSTARLARALSPAF) were selected for comparative confocal microscopy analysis to the same CPP bearing lysosomal targeting sequences (LYSTAT- **CSEWA**YGRKKRRQRRR; LYSPenetratin-**SLLKGRQGIY**RQIKIWFQNRRMKWKK; LYSNicastrin- **SLLKGRQGIY**RLPRCVRSTARLARALSPAF) to assess whether Localization to the lysosome was increased by the inclusion of lysosomal targeting sequences. A control CPP consisting of FITC- **GGGGGGGGGG** was used to establish background signal intensity for the Green channel. Confluent B3 and ARPE19 Cells seeded on coverslips were treated with 1 μM of each peptide in 1 mL of media in a 6 well dish for 2 hours at 37°C. The cells were then washed 3 times in PBS before being incubated with LysoTracker^™^ Red DND-99 according to the manufacture’s instructions. The cells were fixed with 4% Paraformaldehyde in PBS for 10 minutes at room temperature. The nuclear fraction was stained blue using a 1:5000 dilution of Hoescht stain in PBS. The microscope slides were imaged on Leica SP8 confocal microscope utilizing 63X oil immersion objective with UV laser 405nm, VIS laser 488nm, and VIS laser 638.

## Data Availability

Data and materials are available on request for review.

## Data Availability Statement (Required):

All data supporting the findings of this study are available within the paper and its Supplementary Information.
